# Visual processing and interference performance influences on knee angular impulse in ACLR individuals: a cognitive-biomechanical analysis of drop-jumps

**DOI:** 10.1007/s00402-026-06353-w

**Published:** 2026-05-16

**Authors:** Keven Santamaria-Guzman, Hillary Holmes, Jerad Kosek, Brandon Peoples, Kenneth Harrison, Silvia Campos-Vargas, Wendi Weimar, Kristina Neely, Francisco Siles-Canales, Jaimie Roper

**Affiliations:** 1 School of Kinesiology, Auburn University, Auburn, United States; 2 Track and Field, High Point University, High Point, United States; 3 School of Health Sciences, University of Evansville, Evansville, United States; 4 Human Movement Science Research Center, University of Costa Rica, San José, Costa Rica

**Keywords:** Neuro-motor control, Anterior cruciate ligament, Drop-Jump, Knee angular impulse, Biomechanics, Cognition

## Abstract

**Introduction:**

Visual processing speed and cognitive interference control are critical for athletic performance and Anterior Cruciate Ligament (ACL) injury risk. However, how these cognitive functions relate to biomechanical performance after Anterior Cruciate Ligament Reconstruction (ACLR) remains unclear. This gap is clinically relevant given persistent re-injury rates of 20–25% in young athletes returning to sport, suggesting that rehabilitation may insufficiently address cognitive demands. This study examined cognitive differences between ACLR individuals and controls during drop-jump tasks, and their relationship with knee angular impulse.

**Materials and methods:**

Thirty-two females (16 ACLR, 16 controls; 20 ± 1 years) completed cognitive assessments including the Stroop Color and Word Test, Trail Making Test, Digit Span Memory Test, and visual/auditory reaction time tests. Participants performed drop-jumps under four conditions: standard, choice, visual-cued, and audio-cued. Knee angular impulse was calculated for eccentric, concentric, and net phases. Binomial logistic regression identified cognitive predictors distinguishing groups, followed by factorial ANOVA to assess biomechanical differences. Spearman correlations examined relationships between cognition and knee angular impulse.

**Results:**

Three cognitive variables distinguished groups: cognitive interference score, visual simple reaction time, and visual complex reaction time (χ²(3) = 55.090, *p* < 0.001). The ACLR group demonstrated faster visual reaction times but impaired interference control compared to controls. Biomechanically, ACLR participants showed lower eccentric knee angular impulse (*p* = 0.003, d=-0.37), indicating persistent protective strategies. Audio-cued conditions elicited higher eccentric impulse than standard and choice conditions. Minimal correlations between cognitive variables and eccentric knee angular impulse suggest independent (parallel) adaptations.

**Conclusions:**

ACLR individuals exhibit distinct cognitive and biomechanical profiles characterized by enhanced visual reactivity, reduced interference control, and altered landing mechanics. The lack of association between domains indicates parallel adaptations, supporting rehabilitation approaches that independently target both cognitive function and biomechanical performance.

## Introduction

The role of cognitive processes is increasingly recognized for its importance in athletic movements, particularly in tasks requiring rapid decision-making and direction changes. These cognitive processes are essential in motor planning and execution [1, 2]. Critical factors such as reaction time, processing speed, and adaptability to visual stimuli have been linked to injury risk, with slower cognitive responses associated with a higher likelihood of injury [3, 4].

Notably, cognitive abilities have emerged as significant for understanding injuries such as Anterior Cruciate Ligament (ACL) tears. Research has shown that athletes who suffer non-contact ACL injuries often exhibit longer reaction times, slower visual processing speeds, and lower memory scores [5–7]. ACL injury treatment options include surgical reconstruction and rehabilitation, with costs ranging from $20,000 to $50,000 per case. With 100,000–200,000 ACL ruptures annually in the US, the total yearly cost reaches $2–10 billion [8]. Understanding the role of cognitive function in shaping motor behaviors is crucial for developing effective strategies and potentially reducing the incidence of ACL injury. Despite advances in surgical techniques and rehabilitation protocols, approximately 20–25% of young athletes sustain a secondary ACL injury upon returning to sport [9, 10]. This re-injury burden highlights a critical gap: current return-to-sport criteria rely predominantly on physical benchmarks (e.g., strength symmetry, hop tests) while systematically underemphasizing the cognitive demands intrinsic to sport participation. Identifying modifiable cognitive-motor factors that distinguish individuals with ACLR from Non-ACLR peers may therefore provide actionable targets for more comprehensive rehabilitation protocols.

For Individuals with ACL injuries, impaired cognitive abilities can adversely affect motor planning, coordination, and reaction times, which in turn influence how the body responds to knee moments during dynamic activities [7, 11]. For instance, decreased cognitive performance can lead to slower responses to sudden changes in movement or direction, increasing the risk of improper knee joint alignment and heightened mechanical stress.

This relationship underscores that lower cognitive performance can compromise the effectiveness of neuromuscular control, making individuals more susceptible to excessive knee moments and potentially exacerbating the risk of injury or re-injury [9, 10]. However, limited research explores how cognitive processes interact with biomechanics in those who have undergone ACL reconstruction (ACLR). Post-injury, the body often modifies movement patterns due to changes in sensorimotor control, which may also affect cognitive processing as the brain integrates new sensory feedback [12].

In the same way, drop-jump tasks are particularly relevant for ACL injury research as they replicate the high-impact landing mechanics commonly associated with non-contact ACL injuries during deceleration and change-of-direction movements in sport [13–15]. These tasks allow systematic examination of neuromuscular control strategies under varying cognitive demands while maintaining ecological validity for sport-related injury mechanisms [16].

This study aims to investigate cognitive performance and biomechanical characteristics in individuals with ACLR compared to controls during multiple drop-jump tasks. Additionally, it seeks to explore the relationship between cognitive function and knee angular impulse (KAI) differences between groups. We hypothesize that: (1) specific cognitive functions will significantly distinguish individuals who have undergone ACLR from controls, (2) significant differences in KAI will exist between ACLR and control groups, and (3) these cognitive and biomechanical differences will be related, reflecting integrated neuromuscular adaptations following ACL reconstruction. This study contributes to the existing literature by elucidating the relationship between cognitive function and biomechanical performance post-ACLR, with potential implications for rehabilitation strategies and performance protocols.

## Materials and methods

### Participants

Thirty-two females participated in this study (16 ACLR, 16 Control). The ACLR group (age = 20.31 ± 1.70 years, height = 1.69 ± 0.06 m, mass = 67.54 ± 9.10 kg) and Control group (CTRL) (age = 20.38 ± 1.09 years, height = 1.67 ± 0.07 m, mass = 62.98 ± 8.79 kg) were well-matched with no significant differences in age (t(30) = −0.124, *p* = 0.902), height (t(30) = 0.596, *p* = 0.556), or mass (t(30) = 1.443, *p* = 0.159). All participants were comfortable jumping from a 1 ft box. Of ACLR participants, 12 had a single tear, while 4 had multiple contralateral tears (2 double, 2 triple). All underwent reconstruction using various grafts (8 patellar, 5 hamstring, 1 gracilis, 1 artelon synthetic, 1 unknown), This is consistent with the heterogeneous graft profiles reported in prior ACLR biomechanical studies [17]. Participants had their last ACL tear 32.81 ± 15.77 months ago, completed 6.88 ± 3.18 months of rehabilitation, and had at least 6 months post-return-to-play clearance. Exclusion criteria were based on the PAR-Q assessments [18]. Participants were involved in various sports, including Basketball (4), Cross Country (2), Crossfit (1), Gymnastics (1), Lacrosse (2), Running (1), Soccer (8), Softball (2), Tennis and Badminton (2), Track and Field (5) and Volleyball (4). Informed consent was obtained, and the study was approved by the University’s Institutional Review Board. A formal a priori power analysis was not conducted; the final sample of 32 participants (16 per group) represents the total number of eligible individuals recruited within the available resources and timeframe of the study. This constitutes a convenience sample, and the resulting sample size should be considered a limitation when interpreting the statistical power of secondary analyses, particularly the exploratory logistic regression (EPV = 5.3).

### Procedures

#### Surveys

Participants completed an online survey assessing current and past sports participation, injury history, ACL reconstruction details, osteoarthritis status, and concussion history. Data included sport types, participation level, training intensity, number of ACL tears, surgeries, graft types, injury mechanisms, therapy details, and other injuries like sprains and strains.

### Cognitive testing

All cognitive tests were administered consistently by the same researcher in a non-distracting environment. Participants completed the Stroop Color and Word Test, the Trail Making Test A and B (TMT), the Digit Span Memory Test (DS), and computerized simple (SRT) and complex reaction time (CRT) tests for both visual and auditory stimuli.

**Fig. 1:**
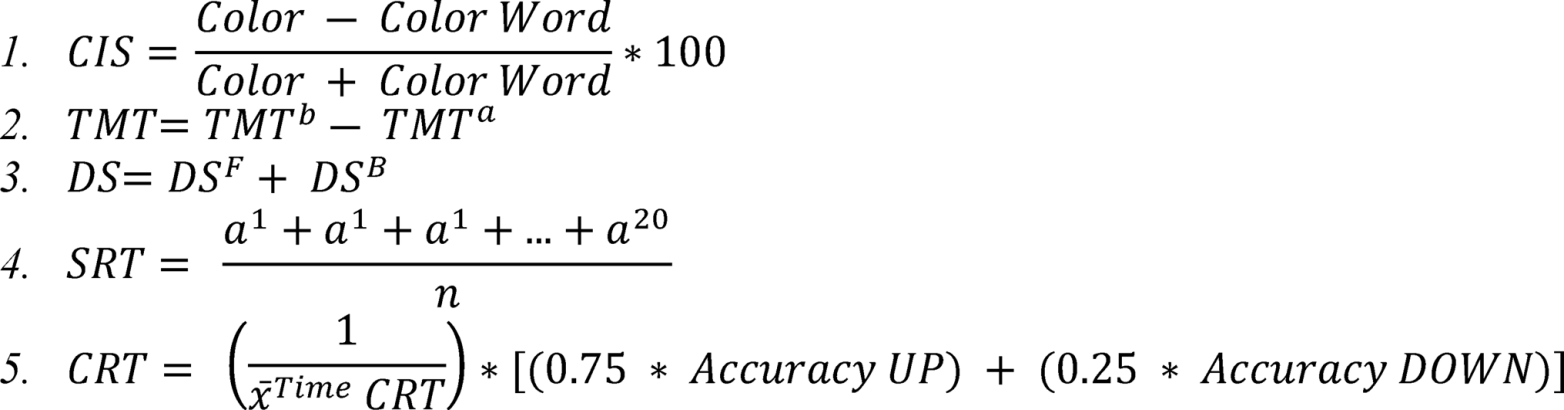
Formulas for Calculating Individual Cognitive Test Scores. Formula 1: Cognitive Interference Score (CIS) derived from the Stroop Color and Word Test, calculated from the number of correct verbal responses within 45 s in color-naming and incongruent (color word) conditions. Formula 2: Trail Making Test (TMT) difference score, calculated as the time difference (in seconds) between Trails B and Trails A performance on the 25-item test. Formula 3: Digit Span (DS) total score, calculated as the sum of the longest sequence correctly recalled in both forward (^F^) and backward (^B^) order. Formula 4: Simple Reaction Time (SRT) average score for both visual (responding to upward-facing triangle with up-arrow key) and auditory (responding to high-pitched horn with up-arrow key) modalities. Formula 5: Complex Reaction Time (CRT) score incorporating both speed and accuracy using an inverse-time weighted approach for visual (responding to upward/downward-facing triangles) and auditory (responding to high/low-pitched horns) modalities, with 75% frequent and 25% rare stimuli presentations

Performance on the Stroop Color and Word Test was quantified by the number of correct verbal responses provided within a 45-second time frame in both the color-naming and incongruent conditions. The cognitive interference score (CIS) was calculated using the formula 1 (Fig. 1) [19–21]. TMT performance was measured by the time (in seconds) taken to complete the 25-item test, with a difference score (Formula 2 in Fig. 1) calculated as the time difference between Trails A and Trails B [22, 23]. For the DS, participants were presented with a series of numbers and instructed to repeat them in both forward and backward order. Scoring was based on the sum of the longest sequence correctly recalled in each direction, as outlined in Formula 3 (Fig. 1) [24].

The SRT and CRT tests were conducted on a laptop. Participants responded to an upward-facing triangle for visual SRT (V-SRT) by pressing the up-arrow key as quickly as possible. In the visual CRT (V-CRT), participants responded to either an upward or downward-facing triangle by pressing the corresponding arrow key as accurately and quickly as possible. For the auditory SRT (A-SRT), participants responded to a high-pitched horn by pressing the up-arrow key as quickly as possible. In the auditory CRT (A-CRT), participants responded to either a high- or low-pitched horn by pressing the up- or down-arrow key as appropriate. In the CRT tests, 75% of trials presented frequent stimuli (upward-facing triangle or high-pitched tone), while 25% presented rare stimuli (downward-facing triangle or low-pitched tone) to create an expectancy manipulation that increased task complexity and cognitive load.

The CRT score incorporates both speed and accuracy using an inverse-time weighted approach (Formula 5, Fig. 1), where higher scores indicate faster reaction times with maintained accuracy. This composite scoring method accounts for speed-accuracy tradeoffs, ensuring that rapid but error-prone responses do not artificially inflate performance scores. Each participant completed all cognitive tests in a single session prior to biomechanical testing. The test battery was administered in fixed order: (1) CIS, (2) TMT A and B, (3) DS, (4) SRT tests (visual then auditory), and (5) CRT tests (visual then auditory). Each SRT condition consisted of 20 trials, and the score was calculate suing the average (Formula 4, Fig. 1), while each CRT condition consisted of 40 trials to ensure adequate presentation of both frequent (30 trials) and rare (10 trials) stimuli. Rest periods of 30–60 s were provided between tests to minimize fatigue. Cognitive testing was completed on the same day as biomechanical testing, with a minimum 15-minute break between sessions.

### Participant preparation

Kinetic data were collected at 1000 Hz with two force plates (AMTI, Watertown, MA), and kinematic data at 100 Hz using a 17-camera motion capture system (Vicon Motion Systems Inc., Oxford, UK). Participants wore 45 markers following Vicon’s Plug-in Gait Full Body Functional Set. Jump cues were triggered by integrating live marker data into MATLAB via Vicon DataStream SDK. Marker data were read in MATLAB at 100Hz, matching the camera frequency. Two markers on the 30 cm jumping platform enabled a MATLAB script to determine its position relative to the participant and capture volume.

#### Protocol

A standardized warm-up protocol included 3 jogging laps (approximately 12 m each) at a self-selected pace, followed by 10 bodyweight squats, double-leg hops, single-leg hops, and 3 countermovement jumps, with adequate rest between activities. Subjects were then instructed and allowed to practice the drop-jump tasks up to 3 times.

For this drop-jump task, subjects jumped forward off a box with both feet, landing simultaneously on bilateral force platforms positioned half their height from the box. Drop jumps required subjects to “jump as high as possible” upon landing, whereas drop lands required a comfortable landing. Trials were repeated if instructions were not followed correctly [25]. Minimal instructions were provided to minimize performance variability due to verbal cues [26], and only up to 4 researchers were present to limit crowd influence [27].

**Fig. 2:**
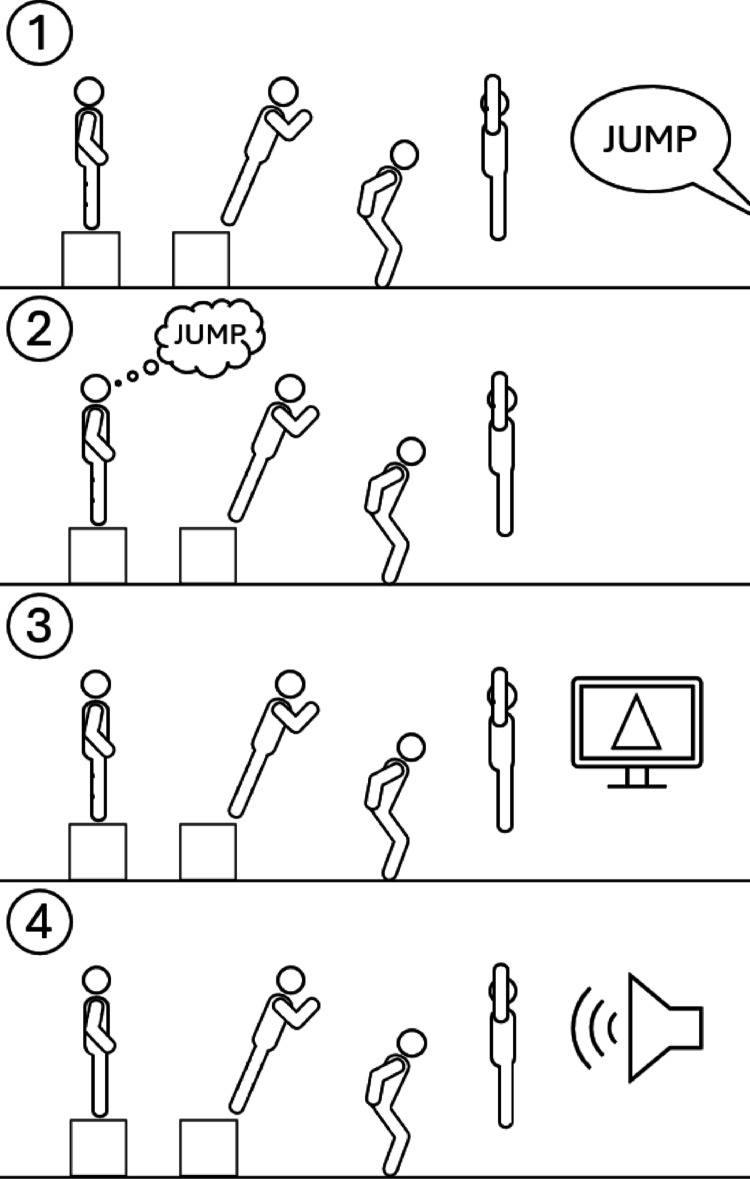
The four drop-jump conditions: (1) Standard, (2) Choice, (**3)** Visual, and **4)** Auditory. Participants jumped forward off a box with both feet, landing simultaneously on bilateral force platforms positioned half their height in four conditions. (1) Standard; participants to jump as high as possible upon landing without additional constraints once they receive the instruction to jump; (2) Choice; participants decided whether to complete a maximal jump or perform a comfortable landing; (3) Visual; probabilistic visual cue condition where an upward-facing triangle appeared on a chest-height screen when the pelvis marker crossed the box edge, signaling required jump completion, with cue absence indicating a landing-only task; (4) Audio; probabilistic auditory cue condition where a horn sound was presented when the pelvis marker crossed the box edge, signaling required jump completion, with cue absence indicating a landing-only task

Four conditions were tested: (1) standard (baseline), (2) choice (volitional decision-making), (3) visual, and (4) audio (external cues) (see Fig. 2 for a schematic representation of the experimental protocol). The visual and audio conditions were designed to reduce motor planning time by providing probabilistic cues when the subject’s pelvis marker crossed the box’s edge. Visual cues appeared as an upward-facing triangle on a chest-height screen, while audio cues utilized a horn sound. Both cue types signaled required jump completion, with cue absence indicating no jump necessary (just box landing task). This methodology aimed to elucidate how reduced planning time through external cueing affects movement execution parameters, particularly those relevant to ACL injury risk factors. Three drop-jumps trials were randomized and not blocked by condition, maintaining cognitive demands. A rest period of 30–60 s was provided between trials, with fatigue monitored using the Borg Rate of Perceived Exertion scale.

### Data reduction

**Fig. 3:**
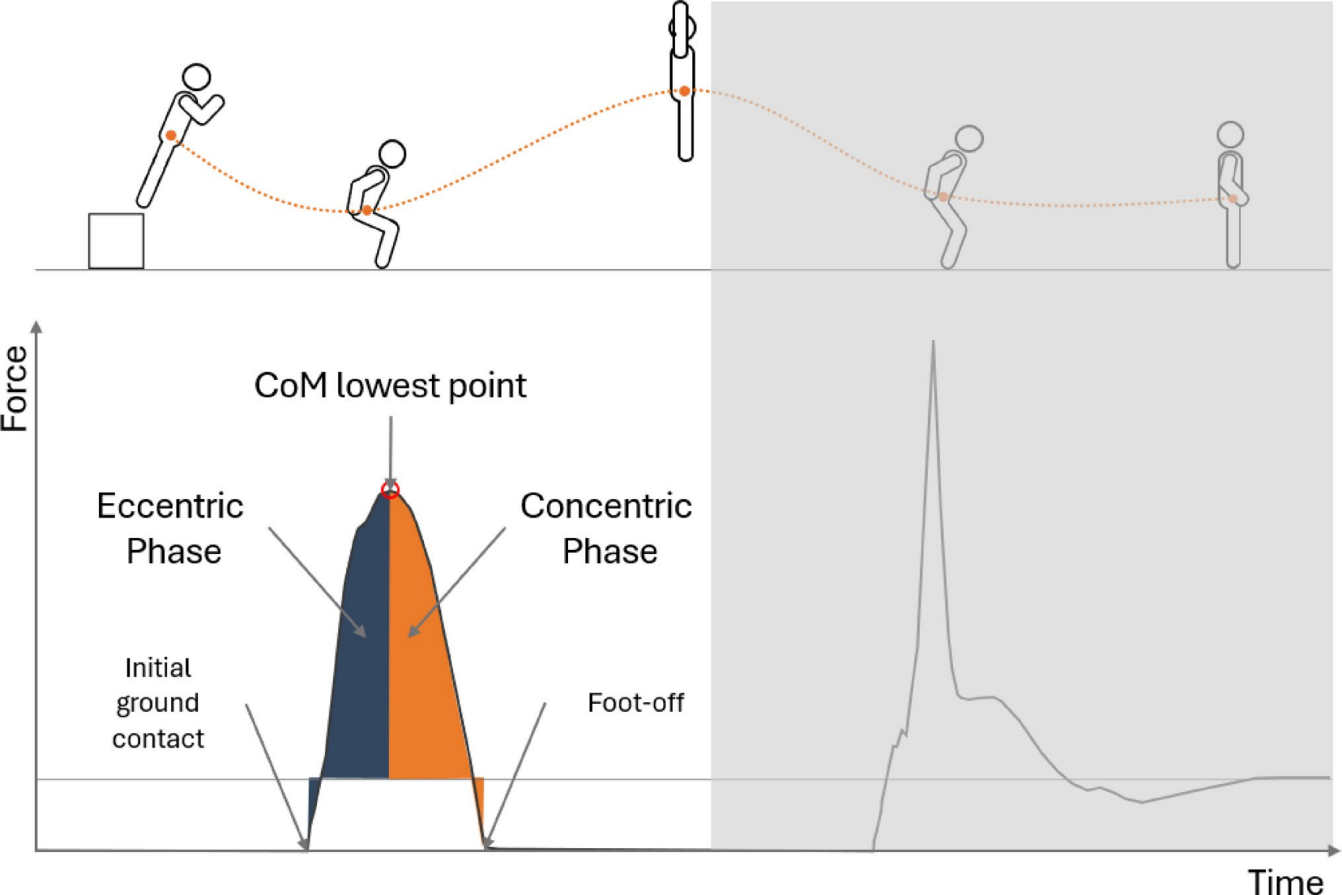
Drop-jump phase identification for knee angular impulse calculation using ground reaction force and center of mass data. The figure illustrates the key temporal landmarks used to calculate the phases of knee angular impulse. Initial ground contact (marked by 5 N force threshold) defines the start of the eccentric phase (navy blue area). The lowest point of the center of mass (CoM) marks the transition between eccentric and concentric phases. Foot-off (marked by force dropping below 5 N threshold) defines the end of the concentric phase (orange area). The red circle clearly distinguishes the CoM lowest point from the initial ground contact marker. KAI^ECC^ eccentric knee angular impulse (ground contact to lowest CoM), KAI^CON^ concentric knee angular impulse (lowest CoM to foot-off), KAI^NET^ net knee angular impulse (algebraic sum of KAI^ECC^ and KAI^CON^).

Force data were filtered using a 4th-order zero-phase low-pass Butterworth filter with a 50 Hz cutoff, consistent with established recommendations for ground reaction force data in impact-loading tasks [28]. Ground contact time was determined using a 5 N force threshold for foot-strike and foot-off. The center of mass (CoM) was determined using Vicon’s Plug-in Gait model. Vertical GRF impulse was calculated by integrating the net vertical ground reaction force with respect to time using the trapezoidal rule throughout each phase [29]. The first landing phase was analyzed for knee angular components. This phase began at initial ground contact (5 N force threshold) and continued through foot-off [30–32]. Knee angular impulse represents the cumulative effect of joint moment over time. In this research, knee angular impulse in the eccentric phase (KAI^ECC^) was calculated from initial ground contact through the lowest point of the CoM, representing the total rotational effect produced as the knee flexes. Knee angular impulse in concentric phase (KAI^CON^) was then calculated from the lowest point of the CoM until foot-off, representing the total rotational effect produced as the knee extends during the take-off portion. The knee angular impulse net (KAI^NET^) was calculated as the algebraic sum of KAI^ECC^ and KAI^CON^, representing the total knee moment generated throughout the entire movement. All KAI calculations used the trapezoidal rule for integration and were normalized to body mass. The three KAI phases are illustrated in Fig. 3.

#### Statistical analysis

All statistical analyses were conducted using JASP 0.95.4 (JASP Team, Amsterdam, Netherlands). To identify cognitive variables that distinguish ACLR from control participants, an exploratory binomial logistic regression was performed with group membership as the dependent variable (Control = 1, ACLR = 0). The initial model incorporated all seven cognitive performance measures (CIS, TMT, DS, A-SRT, A-CRT, V-SRT, and V-CRT). A backward elimination procedure was then applied to identify the most parsimonious model. Given that the resulting model had a modest events-per-variable ratio (EPV = 5.3, below the recommended threshold of 10 for logistic regression), this analysis was treated as exploratory and hypothesis-generating. Model performance was evaluated using multiple fit indices, including chi-square tests and Nagelkerke R². To confirm the robustness of the logistic regression findings, the Mann-Whitney U test was conducted for all cognitive variables to examine individual differences across cognitive tests, with effect sizes calculated using the rank biserial correlations.

Subsequently, separate 2 (Group: ACLR, Control) × 4 (Condition: Standard, Choice, Audio, Visual) factorial analyses of variance (ANOVAs) were conducted for each KAI phase (ECC, CON, NET) to assess biomechanical differences between groups and across task conditions. Levene’s test was used to assess homogeneity of variance across groups and conditions. Visual inspection of Q-Q plots confirmed that the residuals for all three ANOVA models were approximately normal. Levene’s tests confirmed homogeneity of variance across groups and conditions for ECC (F = 0.563, *p* = 0.786), CON (F = 1.058, *p* = 0.391), and NET (F = 0.334, *p* = 0.938). These findings support the appropriateness of parametric ANOVA despite the violation of multivariate normality observed in the correlation analysis. When significant main effects were detected, post-hoc pairwise comparisons with Bonferroni correction were performed to control for Type I error inflation. Effect sizes were calculated using partial eta-squared (η²) for ANOVA main effects and interactions, and Cohen’s d with 95% confidence intervals for pairwise comparisons.

To examine the relationship between cognitive performance and biomechanical measures, we first assessed multivariate normality using the Shapiro-Wilk test. Given that the assumption of multivariate normality was violated (W = 0.921, *p* = 0.001), Spearman’s rank correlation coefficients (rho) were computed between all seven cognitive variables and each of the three KAI phases. The strength of correlations was interpreted using Cohen’s guidelines (small: *r* ≥ 0.10, medium: *r* ≥ 0.30, large: *r* ≥ 0.50). Statistical significance was set at *p* < 0.05 for all analyses. Given the modest EPV of 5.3, the logistic regression findings should be interpreted as exploratory. Future studies replicating this design are encouraged to employ Firth’s penalized-likelihood logistic regression, which reduces finite-sample bias and is particularly recommended when EPV < 10. Similarly, future work would benefit from supplementing frequentist ANOVAs and correlation analyses with Bayesian alternatives, which can quantify the strength of evidence for null findings; a particularly valuable property when interpreting non-significant results such as those for KAI^CON^ and KAI^NET^, where frequentist tests alone cannot distinguish insufficient statistical power from a true null effect.

## Results

### Cognitive performance distinguishes ACLR from control groups

**Fig. 4:**
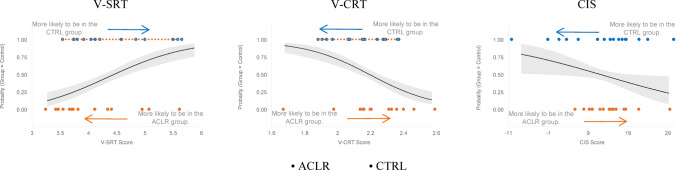
Logistic regression predicting group membership (control vs. ACLR). Blue dots represent Control participants (*n* = 16), orange dots represent ACLR participants (*n* = 16), with the black line showing the logistic prediction curve and gray shading indicating 95% confidence intervals. Arrows indicate the direction of increased likelihood for each group membership. *V-SRT *Visual Simple Reaction Time, *V-CRT * visual complex reaction time, *CIS* cognitive interference score

To identify cognitive variables that distinguish ACLR individuals from matched controls, we conducted an exploratory binomial logistic regression with group membership as the outcome variable. The initial model incorporated all the cognitive performance measures. Following backward elimination, three cognitive measures emerged as significant predictors of group status: CIS, V-SRT, and V-CRT. These variables significantly enhanced the model’s predictive power relative to the null model (χ²(3) = 55.090, *p* < 0.001), demonstrating their collective ability to reliably differentiate between the ACLR and Control groups. The final model accounted for 25.8% of the variance in group status (Nagelkerke R² = 0.258) (see Fig. 4 for group classification performance of the final model).

All three retained cognitive variables exhibited significant predictive capacity for group status. The V-SRT Score (β = 1.205, Odds Ratio = 3.338, z = 5.145, *p* < 0.001) revealed that for each unit increase, the odds of belonging to the Control group (versus the ACLR group) increased by a factor of 3.338, indicating that slower visual simple reaction times were associated with control group membership. Conversely, the V-CRT Score (β = −4.520, Odds Ratio = 0.011, z = −5.025, *p* < 0.001) indicated that each unit increase was associated with a 98.9% decrease in the odds of being in the Control group (1–0.011), suggesting that higher V-CRT scores (indicating faster complex reaction times) were associated with ACLR group membership. Lastly, the CIS (β = −0.038, Odds Ratio = 0.963, z = −2.164, *p* = 0.030) showed that for every unit increase in the interference score, the odds of belonging to the Control group decreased by 3.7% (1–0.963), indicating poorer interference control in the ACLR group.

Given the exploratory nature of this analysis and the modest events-per-variable ratio (EPV = 5.3, below the recommended threshold of 10), being inconclusive [33]. We confirmed these findings using Mann-Whitney U test and a rank biserial r to quantify the magnitude of group differences (Table 1). ACLR participants demonstrated significantly faster V-SRT than controls, with a moderate-to-large effect size. ACLR participants also showed faster V-CRT than controls, indicating a moderate effect. However, ACLR participants exhibited a non-significant poorer interference control compared to controls. These findings indicate that ACLR individuals possess a distinct cognitive profile characterized by enhanced visual reactive capabilities compared to matched controls.

**Table 1:** Descriptive statistics and group comparisons for cognitive performance variables

Cognitive Variable	ACLR	Control	Mann-Whitney U	*p*-value	Rank Biserial *r*
CIS	14.38 ± 5.64	12.61 ± 10.04	8064	0.829	0.016
TMT	21.81 ± 17.13	21.05 ± 17.49	8448	0.666	−0.031
DS	16.63 ± 2.68	17.19 ± 3.79	7776	0.480	0.051
A-SRT	2.62 ± 0.59	2.67 ± 0.35	7936	0.666	0.031
A-CRT	1.67 ± 0.23	1.69 ± 0.13	8896	0.235	−0.086
V-SRT	4.04 ± 0.66	4.48 ± 0.74	4992	< 0.001***	0.391
V-CRT	2.22 ± 0.20	2.14 ± 0.16	10,624	< 0.001***	−0.297

Higher scores indicate better performance for all variables except TMT (where lower scores indicate better executive function performance) and simple reaction times. Rank biserial correlation represents the effect size for Mann-Whitney U tests

*CIS *cognitive interference score, *TMT* trail making test difference score (B-A), *DS* digit span total score, *A-SRT* auditory simple reaction time, *A-CRT* auditory complex reaction time, *V-SRT* visual simple reaction time, *V-CRT* visual complex reaction time

***p < 0.001

### Biomechanical differences between groups

We conducted a 2 (Group: ACLR, Control) × 4 (Condition: Standard, Choice, Visual, Audio) factorial analysis of variance (ANOVA) for each KAI phase. This analysis examined whether group and condition factors independently or interactively influenced knee biomechanics during drop-jump landings.

### Eccentric knee angular impulse

The ANOVA conducted for KAI^ECC^ revealed a significant main effect of Group (F (1, 248) = 8.71, *p* = 0.003, η² = 0.034) and Condition (F (3, 248) = 5.01, *p* = 0.002, η² = 0.057). The interaction between Group and Condition was not significant (F (3, 248) = 0.42, *p* = 0.738, η² = 0.005), indicating that both groups responded similarly to the different task conditions. Levene’s test confirmed homogeneity of variance across groups and conditions (F (7, 248) = 0.56, *p* = 0.786).

Post-hoc pairwise comparisons with Bonferroni correction indicated that the ACLR group (M = 0.324, SD = 0.078) exhibited significantly lower KAI^ECC^ compared to the Control group (M = 0.350, SD = 0.072). This suggests that ACLR participants generated less KAI^ECC^ during the landing phase across all conditions, representing a small-to-moderate effect size difference (Mean Difference = −0.027, *p* = 0.003, Cohen’s d = −0.37).

Regarding condition effects, post-hoc comparisons revealed that the Auditory-cued condition (M = 0.359, SD = 0.073) produced significantly higher KAI^ECC^ than both the Standard condition (M = 0.318, SD = 0.083; *p* = 0.009, Cohen’s d = 0.57) and the Choice condition (M = 0.322, SD = 0.074; *p* = 0.025, Cohen’s d = 0.51). No significant differences emerged between other condition pairs (all *p* > 0.20).

### Concentric and net knee angular impulse

The analysis of KAI^CON^ revealed non-significant main effects for Group (F(1, 248) = 0.58, *p* = 0.447, η² = 0.002) and Condition (F(3, 248) = 0.03, *p* = 0.993, η² < 0.001). The interaction between Group and Condition was also non-significant (F(3, 248) = 0.38, *p* = 0.768, η² = 0.005). Levene’s test indicated homogeneity of variance (F(7, 248) = 1.06, *p* = 0.391).

**Fig. 5:**
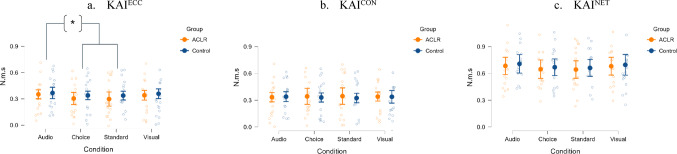
Differential Sensitivity of KAI Phases to Condition Effects in ACLR and Control Participants. Orange circles/SD bars represent ACLR participants (n=16), blue circles/SD bars represent Control participants (n=16). *p < 0.05. Panel (a) Eccentric Knee Angular Impulse (KAI^ECC^) shows significant main effects for Group (F=8.71, p = 0.003, η²=.034; ACLR < Control, Cohen's d=-0.37) and Condition (F=5.01, p = 0.002, η²=.057; Audio > Standard, p = 0.009, d=0.57; Audio > Choice, p = 0.025, d=0.51), with no significant interaction (p = 0.738). Panel (b) Concentric Knee Angular Impulse (KAI^CON^) shows no significant effects for Group (p = 0.447), Condition (p = 0.993), or interaction (p = 0.768). Panel (c) Net Knee Angular Impulse (KAI^NET^) shows no significant effects for Group (p = 0.231), Condition (p = 0.158), or interaction (p = 0.998). Results indicate ACLR individuals exhibited persistent reductions in eccentric knee loading across all conditions, while auditory cueing increased eccentric control in both groups. *N.m.s* Newton-meters per second normalized to body mass

For KAI^NET^, the ANOVA revealed non-significant main effects for Group (F(1, 248) = 1.44, *p* = 0.231, η² = 0.006) and Condition (F(3, 248) = 1.75, *p* = 0.158, η² = 0.021). The interaction between Group and Condition was not significant (F(3, 248) = 0.01, *p* = 0.998, η² < 0.001).(Fig. 5)

### Relationship between cognitive performance and biomechanical measures

To examine whether cognitive performance is related to biomechanical outcomes, we computed Spearman’s rank correlation coefficients (W = 0.921, *p* = 0.001) between the seven cognitive variables and each KAI phase across all participants (Table 2).

**Table 2:** Spearman rank correlations between cognitive variables and knee angular impulse phases

	KAI^ECC^		KAI^CON^		KAI^NET^	
	*r*	*p*	*r*	*p*	*r*	*p*
CIS	0.016	0.803	−0.107	0.089	−0.052	0.404
TMT	−0.028	0.655	−0.021	0.733	−0.036	0.562
DS	0.144	0.021*	0.027	0.668	0.125	0.046*
A-SRT	−0.231	< 0.001***	−0.073	0.242	−0.170	0.007**
A-CRT	0.096	0.126	0.285	< 0.001***	0.219	< 0.001***
V-SRT	0.098	0.118	0.169	0.007**	0.152	0.015*
V-CRT	−0.106	0.092	0.123	0.05*	0.009	0.887

*p < 0.05, **p < 0.01, ***p < 0.001

Critically, the three cognitive variables that distinguished groups in the logistic regression (V-SRT, V-CRT, CIS) showed minimal and non-significant correlations with KAI^ECC^. This pattern suggests that the cognitive measures distinguishing ACLR from control groups and eccentric knee biomechanics represent largely independent constructs. Furthermore, the absence of significant Group × Condition interactions across all KAI phases (all *p* > 0.74) indicates that ACLR and control participants responded similarly to varying task demands despite their distinct cognitive profiles.

These findings suggest that while ACLR individuals exhibit both cognitive and biomechanical differences compared to controls, these differences are consistent with a preliminary framework of largely independent, parallel adaptations that warrant replication before definitive conclusions are drawn. The near-zero correlations between the group-distinguishing cognitive variables and the group-differentiating biomechanical measure (KAI^ECC^) provide strong evidence for this dissociation. This implies that cognitive and motor control adaptations following ACL reconstruction may occur through separate, albeit concurrent, mechanisms.

## Discussion

This study investigated the impact of cognitive performance on motor control during multiple drop-jump tasks in individuals with ACLR and healthy controls. Our findings revealed several key points that contribute to our understanding of both cognitive and biomechanical aspects of ACL injury: (a) Cognitive performance effectively differentiated between groups, with better performance on visual reaction time tasks (both simple and complex) and poorer cognitive interference control characterizing the ACLR group, highlighting a distinct neurocognitive profile among individuals with prior ACL reconstruction [5–7]. (b) ACLR individuals demonstrated consistently lower KAI^ECC^ during landing across all task conditions, suggesting persistent protective motor strategies that remain even after returning to sport. And (c) Audio-cued jump conditions produced significantly greater KAI^ECC^ compared to planned conditions in both groups, indicating that external auditory cueing enhances eccentric knee control during landing, an observation with potential implications for rehabilitation exercise design. Furthermore, KAI^ECC^ exhibited sensitivity to both Group and Condition effects, with ACLR participants consistently generating lower impulses across all task conditions, while KAI^CON^ and KAI^NET^ remained relatively consistent across groups and conditions, suggesting that biomechanical adaptations in this ACLR sample are phase-specific rather than global.

A critical and theoretically significant finding of this study is that cognitive and biomechanical differences in ACLR individuals appear to represent a preliminary explanatory framework consistent with parallel rather than integrated adaptations. The three cognitive variables that distinguished the groups (V-SRT, V-CRT, CIS) showed minimal, non-significant correlations with KAI^ECC^, the only biomechanical measure that showed group differences. Furthermore, the absence of significant interactions across all KAI phases (all *p* > 0.74) indicates that ACLR and control participants responded similarly to varying task demands despite their distinct cognitive profiles. This dissociation may suggest that ACL reconstruction may trigger adaptations in multiple systems, cognitive processing, and motor control, through separate, albeit concurrent, mechanisms rather than through a single integrated pathway. From a rehabilitation perspective, this finding implies that cognitive and biomechanical interventions may need to target these systems independently rather than assuming that improvements in one domain will automatically transfer to the other.

The differentiated cognitive profile observed in ACLR participants reveals a complex adaptation pattern in neurocognitive function. While the ACLR group demonstrated superior performance in both simple and complex visual reaction time tasks, they showed impaired cognitive interference control. This pattern of enhanced reactive capabilities alongside reduced interference control suggests potential compensatory mechanisms in the central nervous system following ACL injury. Previous studies have typically reported global cognitive deficits in ACLR populations [3, 5, 34, 35], making our finding of enhanced reaction times particularly noteworthy. This enhancement might reflect neural reorganization following injury, potentially as an adaptation to maintain rapid response capabilities despite altered proprioceptive feedback. However, the increased susceptibility to cognitive interference could indicate a trade-off in attentional resources, where improved reactive speed comes at the cost of reduced ability to filter irrelevant information, similar to findings in other injury adaptation contexts [12, 36].

On the other hand, examining the biomechanical aspects of our findings, the observed reduction in KAI^ECC^ during landing in ACLR individuals provides insight into long-term movement adaptations following reconstruction. This decreased eccentric loading suggests a persistent protective strategy, even in individuals who have completed rehabilitation and returned to sport. Similar protective mechanisms have been documented in previous studies [37–39], but our findings specifically identify the eccentric phase as the target of this adaptation. The selective nature of this modification (occurring only during the eccentric phase without significant changes in concentric or net impulse) suggests a sophisticated neural control strategy rather than global movement inhibition. This specificity might represent an unconscious optimization between protecting the reconstructed ligament and maintaining functional performance [5, 7, 12, 25, 34, 35, 40].

Further analysis of the sensorimotor aspects revealed that audio-cued jumps elicited increased eccentric knee control compared to planned jumps, providing important insights about sensorimotor integration in dynamic tasks. This enhancement of eccentric control under audio cueing may reflect not only the influence of sensory modality but also the temporal and rhythmic properties of auditory cues. Recent evidence suggests that the temporal structure of auditory cues, particularly rhythmic properties, plays a critical role in regulating movement timing and neuromuscular coordination during landing tasks [41]. The additional processing time required for auditory stimuli, combined with their temporal structure, might facilitate more complete motor planning and enhance eccentric control mechanisms. This finding aligns with recent work showing that slower processing can sometimes lead to more controlled movement execution [25].

Given these findings, the role of cognitive function emerges as a critical consideration in ACL injury risk and rehabilitation. The distinctive cognitive profile observed in ACLR participants (characterized by superior reaction times but impaired interference control) may represent more than just a post-injury adaptation. This pattern could potentially identify individuals at higher risk for non-contact ACL injuries, particularly in situations requiring sustained attention amid distractions, which is common in sport environments. Recent studies have shown that neurocognitive deficits precede and may predict ACL injury risk [3–6, 34], with specific impairments in visuospatial attention and processing speed increasing injury odds by up to 3-fold [7, 42]. The relationship between cognitive processing and movement control aligns with emerging evidence that decreased neurocognitive performance correlates with higher-risk biomechanical patterns during dynamic tasks [4, 5, 11, 35]. Furthermore, studies have demonstrated that athletes with lower cognitive performance scores show decreased dynamic postural control and increased landing forces, particularly during dual-task conditions [43–45].

The parallel nature of cognitive and biomechanical adaptations has important clinical implications for ACL rehabilitation. Current rehabilitation protocols typically focus predominantly on restoring physical function, with cognitive factors receiving less systematic attention. Our findings suggest that rehabilitation programs should incorporate both cognitive training (e.g., improving interference control, enhancing rapid decision-making under pressure) and biomechanical retraining (e.g., enhancing landing mechanics, increasing eccentric knee loading capacity) as complementary rather than redundant components [12, 34]. The dissociation between cognitive and biomechanical measures implies that addressing movement patterns alone may not resolve cognitive adaptations, and vice versa. Specifically, interventions targeting visual processing speed and interference control, perhaps through sport-specific reactive drills, dual-task training, or neurocognitive exercises, may be warranted alongside traditional strength and movement retraining [36]. Additionally, the enhanced reactive capabilities observed in ACLR individuals, while potentially compensatory, could be leveraged as a strength in return-to-sport programming if appropriately channeled [6, 9, 34].

While this study provides novel insights through its comprehensive assessment of both cognitive and biomechanical parameters across multiple jump conditions, certain limitations must be considered when interpreting these results. First, the logistic regression analysis, while revealing meaningful cognitive distinctions between groups, had a modest events-per-variable ratio (EPV = 5.3, below the recommended 10), being inconclusive [33] and should therefore be interpreted as exploratory and hypothesis-generating rather than definitive. The confirmation of these findings through independent statistical tests (Mann-Whitney U tests and effect size calculations) provides additional confidence, but replication in larger samples is needed. However, considering our sample size, future studies would include larger samples to confirm null findings, particularly for KAI^CON^ and KAI^NET^, where no group differences emerged. To address the small EPV limitation, future studies are encouraged to employ Firth’s penalized likelihood logistic regression, which reduces finite-sample bias, and to supplement frequentist tests with Bayesian analyses that can quantify the strength of evidence for null findings rather than merely failing to reject the null hypothesis.

Second, our sample exhibited heterogeneity in injury characteristics, including variation in the number of ACL tears (12 single tears, 4 multiple tears), graft types (patellar, hamstring, gracilis, synthetic), time since surgery (range: 17–64 months, M = 32.81 ± 15.77 months), and sport backgrounds (10 different sports). While this heterogeneity reflects the diversity of real-world ACLR populations, consistent with similarly heterogeneous samples in prior biomechanical research [17], it likely increased within-group variance and reduced the precision of our estimates. Graft-specific differences in proprioceptive restoration and neuromuscular control may have produced divergent adaptations that are obscured in a pooled ACLR analysis. Future research with larger, more homogeneous samples could clarify whether specific injury or surgical characteristics moderate the cognitive-biomechanical relationships observed here.

Third, the cross-sectional design precludes determination of whether the observed cognitive and biomechanical differences preceded injury, resulted from injury and reconstruction, or reflect ongoing compensatory adaptations. Longitudinal research tracking individuals from pre-injury through return-to-sport could clarify the temporal relationships and causal mechanisms underlying these observations. Additionally, while we carefully controlled for time since return-to-sport (minimum 6 months), participants were not formally matched by sport type or competitive level, which may have contributed to heterogeneity in both cognitive and biomechanical performance.

Fourth, although our findings suggest parallel rather than integrated cognitive-biomechanical adaptations, we cannot rule out the possibility that more complex, non-linear relationships exist that were not captured by correlation analyses. Advanced analytical approaches such as machine learning or dynamical systems analysis might reveal subtle interactions between cognitive and motor systems that are not apparent in traditional statistical frameworks.

Based on these findings and limitations, several key research directions warrant investigation. Longitudinal studies are urgently needed to examine whether the observed cognitive profile represents a pre-existing risk factor for ACL injury or develops as a consequence of injury. This aligns with recent calls for prospective studies investigating cognitive function as a predictor of injury risk [35]. Additionally, investigation of targeted interventions incorporating both cognitive and motor training could help optimize injury prevention strategies, particularly given evidence that dual-task training can improve both cognitive performance and movement control [46, 47]. Moreover, examination of these cognitive-motor interactions under more complex, sport-specific conditions would enhance ecological validity and clinical applicability, potentially leading to more effective screening tools for injury risk. Future research should also explore the development of cognitive training protocols specifically designed to enhance interference control while maintaining quick reaction times, as this combination appears particularly relevant to injury risk and prevention.

## Conclusions

This study provides novel evidence that individuals with ACLR exhibit distinct cognitive and biomechanical adaptations that develop in parallel rather than through integrated mechanisms. Cognitive variables (V-SRT, V-CRT, CIS) successfully distinguished ACLR from controls, with ACLR individuals showing enhanced visual reactivity alongside impaired interference control. Biomechanically, they demonstrated persistent reductions in eccentric knee angular impulse during landing, suggesting protective strategies that remain post–return to sport. Critically, minimal correlations between cognitive and biomechanical variables, and the absence of interactions, indicate these represent independent adaptations rather than causally linked processes. Given the modest sample size and within-group heterogeneity, this framework should be considered hypothesis-generating.

These findings have important implications for rehabilitation and injury prevention. The independence of adaptations suggests protocols should target cognitive and biomechanical systems separately. Interventions combining interference control training with biomechanical retraining to optimize eccentric loading may be more effective than assuming cross-domain transfer. Future research should evaluate dual-domain approaches and clarify whether the observed cognitive profile reflects pre-injury risk or post-injury adaptation. Clinically, return-to-sport decisions should include assessment of interference control and visual processing speed alongside physical benchmarks, as both appear to be independently necessary targets for comprehensive rehabilitation.

## Data Availability

The dataset used and analyzed during the current study is available from the corresponding author on reasonable request.

## References

[1] Mejane J, Faubert J, Romeas T, Labbe DR (2019) The combined impact of a perceptual–cognitive task and neuromuscular fatigue on knee biomechanics during landing. Knee 26(1):52–60 30583887 10.1016/j.knee.2018.10.017

[2] Scharfen H-E, Memmert D (2019) The relationship between cognitive functions and sport-specific motor skills in elite youth soccer players. Front Psychol 10:817 31105611 10.3389/fpsyg.2019.00817 PMC6494938

[3] Swanik CB (2015) Brains and sprains: the brain’s role in noncontact anterior cruciate ligament injuries. J Athl Train 50(10):1100–1102 26340611 10.4085/1062-6050-50.10.08 PMC4641549

[4] Wilke J, Groneberg D, Banzer W, Giesche F (2020) Perceptual–cognitive function and unplanned athletic movement task performance: a systematic review. Int J Environ Res Public Health 17(20):7481 33066649 10.3390/ijerph17207481 PMC7602452

[5] Bertozzi F, Fischer PD, Hutchison KA, Zago M, Sforza C, Monfort SM (2023) Associations between cognitive function and ACL injury-related biomechanics: a systematic review. Sports Health 15(6):855–866 36680310 10.1177/19417381221146557 PMC10606969

[6] Gokeler A, Benjaminse A, Della Villa F, Tosarelli F, Verhagen E, Baumeister J (2021) Anterior cruciate ligament injury mechanisms through a neurocognition lens: implications for injury screening. BMJ open sport and exercise medicine 7(2):e001091 10.1136/bmjsem-2021-001091 PMC8130757 34055386

[7] Chaput M, Onate JA, Simon JE, Criss CR, Jamison S, McNally M et al (2022) Visual cognition associated with knee proprioception, time to stability, and sensory integration neural activity after ACL reconstruction. J Orthop Res 40(1):95–104 33620108 10.1002/jor.25014

[8] Su AW, Saboe M, Condliffe S, Hazen K, Atanda A, Rogers K (2024) Adolescent anterior cruciate ligament reconstruction: a decade of rising surgical cost. Am J Manag Care 30(6):e178–e183 38912932 10.37765/ajmc.2024.89565

[9] Wiggins AJ, Grandhi RK, Schneider DK, Stanfield D, Webster KE, Myer GD (2016) Risk of secondary injury in younger athletes after anterior cruciate ligament reconstruction: a systematic review and meta-analysis. Am J Sports Med 44(7):1861–1876 26772611 10.1177/0363546515621554 PMC5501245

[10] Beischer S, Gustavsson L, Senorski EH, Karlsson J, Thomeé C, Samuelsson K et al (2020) Young athletes who return to sport before 9 months after anterior cruciate ligament reconstruction have a rate of new injury 7 times that of those who delay return. J Orthop Sports Phys Ther 50(2):83–90 32005095 10.2519/jospt.2020.9071

[11] Giesche F, Wilke J, Engeroff T, Niederer D, Hohmann H, Vogt L et al (2020) Are biomechanical stability deficits during unplanned single-leg landings related to specific markers of cognitive function? J Sci Med Sport 23(1):82–88 31628001 10.1016/j.jsams.2019.09.003

[12] Needle AR, Lepley AS, Grooms DR (2017) Central nervous system adaptation after ligamentous injury: a summary of theories, evidence, and clinical interpretation. Sports Med 47:1271–1288 28005191 10.1007/s40279-016-0666-y

[13] Ortiz A, Olson S, Libby CL, Trudelle-Jackson E, Kwon YH, Etnyre B et al (2008) Landing mechanics between noninjured women and women with anterior cruciate ligament reconstruction during 2 jump tasks. Am J Sports Med 36(1):149–157. 10.1177/0363546507307758 17940142 10.1177/0363546507307758 PMC2744382

[14] Imai S, Harato K, Morishige Y, Kobayashi S, Niki Y, Sato K et al (2022) Effects of dual task interference on biomechanics of the entire lower extremity during the drop vertical jump. J Hum Kinet 81:5–14. 10.2478/hukin-2022-0001 35291634 10.2478/hukin-2022-0001 PMC8884875

[15] Redler LH, Watling JP, Dennis ER, Swart E, Ahmad CS (2016) Reliability of a field-based drop vertical jump screening test for ACL injury risk assessment. Phys Sportsmed 44(1):46–52. 10.1080/00913847.2016.1131107 26651526 10.1080/00913847.2016.1131107

[16] Ma B, Zhang T-T, Jia Y-D, Wang H, Zhu X-Y, Zhang W-J et al (2022) Characteristics of vertical drop jump to screen the anterior cruciate ligament injury. European Review for Medical and Pharmacological Sciences. 10.26355/eurrev_202210_30008 10.26355/eurrev_202210_30008 36314309

[17] Cronström A, Tengman E, Häger CK (2023) Return to sports: a risky business? A systematic review with meta-analysis of risk factors for graft rupture following ACL reconstruction. Sports Med 53(1):91–110 36001289 10.1007/s40279-022-01747-3 PMC9807539

[18] Warburton DE, Jamnik VK, Bredin SS, Gledhill N (2011) The physical activity readiness questionnaire for everyone (PAR-Q+) and electronic physical activity readiness medical examination (ePARmed-X+). Health Fit J Can 4(2):3–17

[19] Barbarotto R, Laiacona M, Frosio R, Vecchio M, Farinato A, Capitani E (1998) A normative study on visual reaction times and two Stroop colour-word tests. Ital J Neurol Sci 19:161–170 10933471 10.1007/BF00831566

[20] Scarpina F, Tagini S (2017) The Stroop color and word test. Front Psychol 8:241674 10.3389/fpsyg.2017.00557 PMC5388755 28446889

[21] Valgimigli S, Padovani R, Budriesi C, Leone M, Lugli D, Nichelli P (2010) The Stroop test: a normative Italian study on a paper version for clinical use. G Ital Psicol 37:945–956

[22] Bowie CR, Harvey PD (2006) Administration and interpretation of the Trail Making Test. Nat Protoc 1(5):2277–2281 17406468 10.1038/nprot.2006.390

[23] Sánchez-Cubillo I, Periáñez JA, Adrover-Roig D, Rodríguez-Sánchez JM, Ríos-Lago M, Tirapu J et al (2009) Construct validity of the Trail Making Test: role of task-switching, working memory, inhibition/interference control, and visuomotor abilities. J Int Neuropsychol Soc 15(3):438–450 19402930 10.1017/S1355617709090626

[24] Iverson GL, Franzen MD (1994) The Recognition Memory Test, digit span, and Knox Cube Test as markers of malingered memory impairment. Assessment 1(4):323–334

[25] Holmes HH, Talmage JLD, Neely KA, Roper JA (2023) Cognitive demands influence drop jump performance and relationships with leg stiffness in healthy young adults. J Strength Cond Res 37(1):74–83 36515592 10.1519/JSC.0000000000004178

[26] Brinkerhoff SA, Murrah WM, Hutchison Z, Miller M, Roper JA (2022) Words matter: instructions dictate self-selected walking speed in young adults. Gait Posture 95:223–226 31395467 10.1016/j.gaitpost.2019.07.379

[27] Friesen KB, Zhang Z, Monaghan PG, Oliver GD, Roper JA (2020) All eyes on you: how researcher presence changes the way you walk. Sci Rep 10(1):17159 33051502 10.1038/s41598-020-73734-5 PMC7554041

[28] Winter DA (2009) Biomechanics and motor control of human movement. John wiley and sons

[29] Moir GL, Snyder BW, Connaboy C, Lamont HS, Davis SE (2018) Using drop jumps and jump squats to assess eccentric and concentric force-velocity characteristics. Sports 6(4):125 30352975 10.3390/sports6040125 PMC6316443

[30] Harry JR, Paquette MR, Schilling BK, Barker LA, James CR, Dufek JS (2018) Kinetic and electromyographic subphase characteristics with relation to countermovement vertical jump performance. J Appl Biomech 34(4):291–297 29485344 10.1123/jab.2017-0305

[31] Mundy PD, Smith NA, Lauder MA, Lake JP (2017) The effects of barbell load on countermovement vertical jump power and net impulse. J Sports Sci 35(18):1781–1787 10.1080/02640414.2016.1236208 28282758

[32] Peng H-T (2011) Changes in biomechanical properties during drop jumps of incremental height. J Strength Cond Res 25(9):2510–2518 21869631 10.1519/JSC.0b013e318201bcb3

[33] van Smeden M, de Groot JAH, Moons KGM, Collins GS, Altman DG, Eijkemans MJC et al (2016) No rationale for 1 variable per 10 events criterion for binary logistic regression analysis. BMC Med Res Methodol 16(1):163. 10.1186/s12874-016-0267-3 27881078 10.1186/s12874-016-0267-3 PMC5122171

[34] Grooms D, Appelbaum G, Onate J (2015) Neuroplasticity following anterior cruciate ligament injury: a framework for visual-motor training approaches in rehabilitation. J Orthop Sports Phys Ther 45(5):381–393 25579692 10.2519/jospt.2015.5549

[35] Wilke J, Groneberg DA (2022) Neurocognitive function and musculoskeletal injury risk in sports: a systematic review. J Sci Med Sport 25(1):41–45 34303619 10.1016/j.jsams.2021.07.002

[36] Reyes MA, Probasco MO, Worby TN, Loertscher DE, Soderbeck LK, Huddleston WE (2022) Lower kinetic chain, meet the thinking brain: a scoping review of cognitive function and lower extremity injury risk. Int J Sports Phys Ther 17(5):787 35949381 10.26603/001c.36814 PMC9340845

[37] An YW, Lobacz AD, Baumeister J, Rose WC, Higginson JS, Rosen J et al (2019) Negative emotion and joint-stiffness regulation strategies after anterior cruciate ligament injury. J Athl Train 54(12):1269–1279 31553654 10.4085/1062-6050-246-18 PMC6922561

[38] Kotsifaki R, Sideris V, King E, Bahr R, Whiteley R (2023) Performance and symmetry measures during vertical jump testing at return to sport after ACL reconstruction. Br J Sports Med 57(20):1304–1310 37263763 10.1136/bjsports-2022-106588

[39] Schmitt LC, Paterno MV, Ford KR, Myer GD, Hewett TE (2015) Strength asymmetry and landing mechanics at return to sport after ACL reconstruction. Med Sci Sports Exerc 47(7):1426 25373481 10.1249/MSS.0000000000000560 PMC4418954

[40] Coutlee CG, Huettel SA (2012) The functional neuroanatomy of decision making: prefrontal control of thought and action. Brain Res 1428:3–12 21676379 10.1016/j.brainres.2011.05.053 PMC3202063

[41] Meng L, Wang Y, Wang Z, Liu Y, Tan Y, Zhang Y et al (2025) Effects of auditory rhythmic adaptation on lower limb joint mechanics during single-leg drop landings in individuals with functional ankle instability. Front Hum Neurosci 19:1579260 40672743 10.3389/fnhum.2025.1579260 PMC12263649

[42] Rikken KTH, Panneman T, Vercauteren F, Gokeler A, Benjaminse A (2024) Increased visual attentional demands alter lower extremity sidestep cutting kinematics in male basketball players. Int J Sports Phys Ther 19(11):1304–1313. 10.26603/001c.124804 39502550 10.26603/001c.124804 PMC11534173

[43] Herman DC, Barth JT (2016) Drop-jump landing varies with baseline neurocognition: implications for anterior cruciate ligament injury risk and prevention. Am J Sports Med 44(9):2347–2353 27474381 10.1177/0363546516657338 PMC6039105

[44] Monfort SM, Pradarelli JJ, Grooms DR, Hutchison KA, Onate JA, Chaudhari AM (2019) Visual-spatial memory deficits are related to increased knee valgus angle during a sport-specific sidestep cut. Am J Sports Med 47(6):1488–1495 30986095 10.1177/0363546519834544

[45] Shibata S, Takemura M, Miyakawa S (2018) The influence of differences in neurocognitive function on lower limb kinematics, kinetics, and muscle activity during an unanticipated cutting motion. Phys Ther Res 21(2):44–52 30697509 10.1298/ptr.E9938 PMC6336436

[46] Fischer PD, Hutchison KA, Becker JN, Monfort SM (2021) Evaluating the spectrum of cognitive-motor relationships during dual-task jump landing. J Appl Biomech 37(4):388–395 34271547 10.1123/jab.2020-0388

[47] Lempke LB, Oh J, Johnson RS, Schmidt JD, Lynall RC (2021) Single-versus dual-task functional movement paradigms: a biomechanical analysis. J Sport Rehabil 30(5):774–785 33494045 10.1123/jsr.2020-0310

